# Distinct temporal dynamics of planktonic archaeal and bacterial assemblages in the bays of the Yellow Sea

**DOI:** 10.1371/journal.pone.0221408

**Published:** 2019-08-26

**Authors:** Jong-Geol Kim, Joo-Han Gwak, Man-Young Jung, Sung-Uk An, Jung-Ho Hyun, Sanghoon Kang, Sung-Keun Rhee

**Affiliations:** 1 Department of Microbiology, Chungbuk National University, Gaeshin-dong, Heungduk-gu, Cheongju, South Korea; 2 Department of Microbiology and Ecosystem Science, Division of Microbial Ecology, University of Vienna, Althanstrasse, Vienna, Austria; 3 Department of Marine Sciences and Convergent Technology, Hanyang University, Hanyangdaehak-ro Ansan, Gyeonggi-do, South Korea; 4 Department of Biological Sciences, Eastern Illinois University, Charleston, IL, United States of America; Fudan University, CHINA

## Abstract

The Yellow Sea features unique characteristics due to strong tides and nutrient-enriched freshwater outflows from China and Korea. The coupling of archaeal and bacterial assemblages associated with environmental factors at two bay areas in the Yellow Sea was investigated. Temporal variations of the archaeal and bacterial assemblages were shown to be greater than the spatial variations based on an analysis of the 16S rRNA gene sequences. Distinct temporal dynamics of both planktonic archaeal and bacterial assemblages was associated with temperature, NO_2_^-^, and chlorophyll a ([chl-*a*]) concentrations in the bays of the Yellow Sea. The [chl-*a*] was the prime predictor of bacterial abundance, and some taxa were clearly correlated with [chl-*a*]. *Bacteroidetes* and *Alpha-proteobacteria* dominated at high [chl-*a*] stations while *Gamma-proteobacteria* (esp. SAR86 clade) and *Actinobacteria* (*Candidatus* Actinomarina clade) were abundant at low [chl-*a*] stations. The archaeal abundance was comparable with the bacterial abundance in most of the October samples. Co-dominance of Marine Group II (MGII) and *Candidatus* Nitrosopumilus suggests that the assimilation of organic nitrogen by MGII could be coupled with nitrification by ammonia-oxidizing archaea. The distinct temporal dynamics of the archaeal and bacterial assemblages might be attributable to the strong tides and the inflow of nutrient-rich freshwater.

## Introduction

Archaea and bacteria actively react to changes in environmental conditions due to their large abundance, great diversity, and fast growth. Archaeal and bacterial assemblages thus provide essential perspectives for understanding the functions of archaea and bacteria in terms of their ecosystem services in marine ecosystems [1, 2].

Bacteria are a major component in microbial food webs and biogeochemical cycles in marine ecosystems [1, 3]. Furthermore, molecular approaches have revealed that archaea, in addition to bacteria, are also abundant planktonic microbial components, and advances in omics approaches have revealed that planktonic archaea have major roles in biogeochemical cycles including the nitrogen and carbon cycles [4–8]. Ammonia-oxidizing archaea, which used to be called Marine Group I, belong to *Thaumarchaeota* and have become increasingly abundant at greater depths [4, 9, 10]. Considerable progress has been made in the physiology, biochemistry, and ecological functions of ammonia-oxidizing archaea belonging to *Ca*. Nitrosopumilus of *Thaumarchaeota* [6, 11, 12]. Three groups of *Euryarchaeota* are less known due to the lack of available isolates in these groups. MGII of *Euryarchaeota* [13] has generally been observed to dominate in archaeal communities of the surface ocean and is predicted to have heterotrophic life styles. However, the mechanisms controlling the distribution of *Archaea* in different water columns of the ocean remain elusive.

The Yellow Sea (YS) features unique oceanographic characteristics. For example, strong currents/tides and nutrient-enriched freshwater outflows from China and Korea contribute to localized phytoplankton blooms along the coastal areas of the YS. Furthermore, the YS is considered one of the most complicated continental sea areas in the world because the seasonal variations of the currents, air temperature, river runoff, and wind stress in this shallow sea generate a high variability in temperature and salinity [14]. Research on the changes in the YS environmental factors during a 25-year period of the last century suggested clear trends for nutrient concentrations [15], which might be associated with industrial and agricultural wastes through run-off and atmospheric deposition into the sea as well as the changing climate [15, 16]. The change in nutrient concentrations and stoichiometry consequently can be associated with the increasing frequency of algal blooms and hypoxic dead zones, which are related to changes in the prokaryote community structure and dynamics [17].

The archaeal and bacterial community composition associated with phytoplankton blooms in the coastal waters of the YS near Korea has not to our knowledge been documented at the taxa-specific level using next generation sequencing. In this study, we carried out temporal water sampling at two bay areas on the west coast of the Korean Peninsula over one year to investigate the archaeal and bacterial assemblages associated with the environmental factors. Distinct archaeal and bacterial assemblages were observed at the bay areas on the west coast of the Korean Peninsula. Dynamic temporal changes in the archaeal and bacterial assemblages were observed with an archaeal bloom composed of *Ca*. Nitrosopumilus and MGII in autumn (October). Environmental factors including temperature, NO_2_^-^, and [chl-*a*] were identified to be associated with changes of the archaeal and bacterial assemblages. The findings contribute to our knowledge of patterns of temporal changes of prokaryotic assemblages in coastal seawaters.

## Materials and methods

### Ethics statement

No specific permissions were required for collection of surface seawater samples from two bays called Garorim and Gyeonggi on the west coast of the Korean Peninsula since these locations are not protected area. We confirm that the field studies did not involve endangered or protected species.

### Site description and sampling

**Fig 1:**
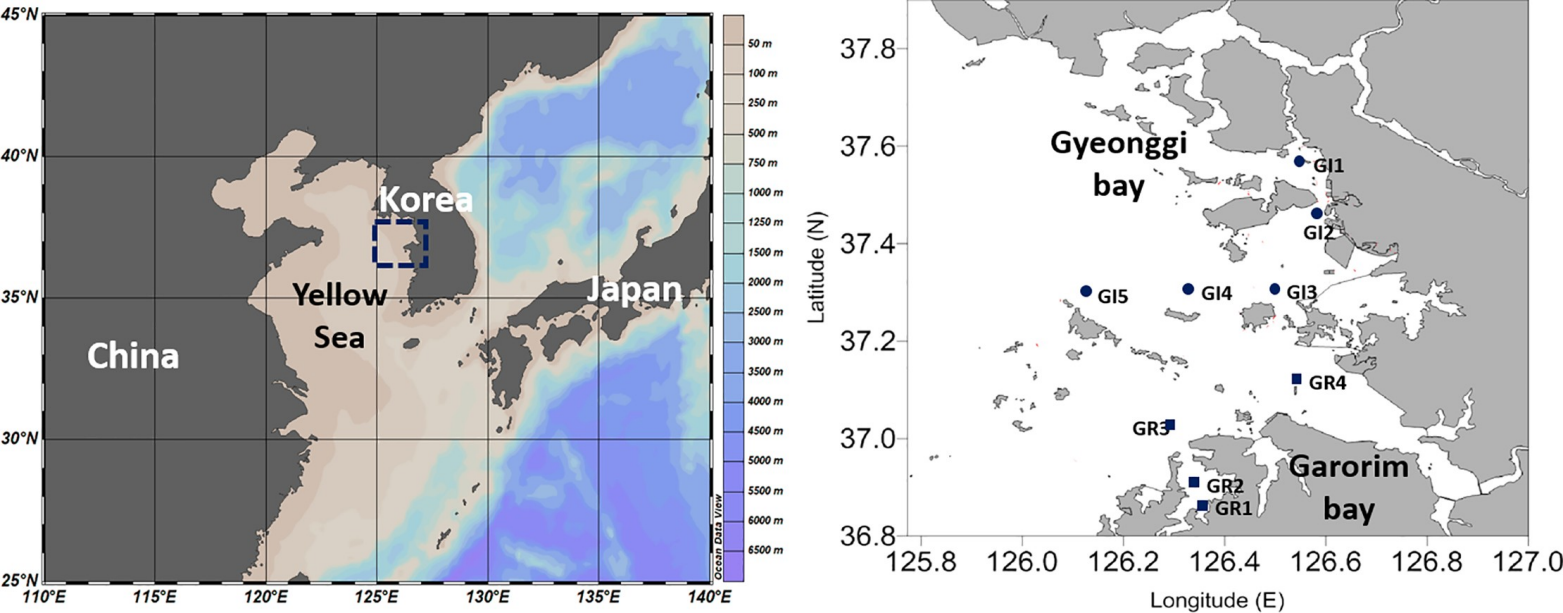
Location of sampling sites in two bay areas in the Yellow Sea.

Surface seawater samples were collected from two bays called Garorim (GR) and Gyeonggi (GI) on the west coast of the Korean Peninsula (Fig 1 and Table A in S1 Appendix) as part of the Korean Long-term Marine Ecological Research Program, which was conducted in April, July, and October of 2015 and in February 2016. The major difference between the two bays is that Garorim Bay has no large inputs of freshwater whereas Gyenonggi Bay is affected by freshwater discharged from the Han River. Each seawater sample (2 L) was filtered immediately through a Whatman GF/A filter (pore size: 1.6 μm, diameter: 45mm) to remove any suspended particles and eukaryotes [18–20] before being filtered through a 0.22 μm pore-size filter (diameter: 45mm, Supor polyethersulfone, Pall Life Sciences) to capture the archaeal and bacterial cells using a vacuum pump. The filters were preserved at -80°C until DNA extraction, which was performed after enzymatic lysis and phenol:chloroform purification [21]. The concentration of DNA was determined using a Nano-Drop ND-1000 spectrophotometer (NanoDrop Technologies, Wilmington, DE).

### Seawater properties

Water temperature, salinity, pH, and dissolved oxygen (DO) were measured *in situ* using a Multi-Parameter Sonde (YSI-6600V2: YSI, Yellow springs, OH, USA). Seawater samples were immediately transferred to the laboratory to analyze other properties of the water. SPM (suspended particulate matter) was determined by filtering the material in a known volume of the sample onto pre-weighed filter papers (GF/F, Whatman, Maidstone, UK). Filters were washed with fresh water and then dried and weighed. Particulate organic carbon (POC) was determined for samples filtered through pre-combusted GF/F filters, which were dried and measured using a CHNS analyzer (Carlo Erba, Val de Reuil, France). The concentrations of the major inorganic nutrients (NH_4_^+^, NO_2_^-^, NO_3_^-^, PO_4_^2-^, and silicate) were analyzed using a Bran and Luebbe model Quatro AA (Auto Analyzer) according to the manufacturer’s instructions. The dissolved organic carbon (DOC) measurements were carried out by a high-temperature catalytic oxidation technique (HTCO), as described in Suzuki, Tanoue [22], using a TOC-VCPH (Shimazu, Kyoto, Japan). The total [Chl-*a*] concentration was determined using a spectrophotometer after extracting the filter with 90% acetone at 4°C for 24h in the dark [23].

### Quantification of bacterial and archaeal 16S rRNA genes

Bacterial and archaeal 16S rRNA gene copies were quantified using a MiniOpticon real-time PCR detection system (BIO-RAD, Hercules, California, USA) and the built-in Opticon Monitor Software version 3.1 (Bio-Rad Laboratories, Hercules, CA, USA). The bacterial and archaeal 16S rRNA genes were amplified using the archaea-specific primer set 519F-727R and the bacteria-specific primer set bac518F-bac786R as described by Park and colleagues [24]. The real-time PCR efficiencies of the bacterial and archaeal 16S rRNA gene assays were 90–95% and 92–96%, respectively, with r^2^ values of ≥ 0.99 in all assays. The following thermal cycling parameters were used to amplify all genes: 15 min. at 95°C followed by 40 cycles of 20 s at 95°C, 20 s at 55°C and 20 s at 72°C, and readings were taken between each cycle. Standard curves were prepared in each run using standards of reference genes (archaeal 16S rRNA gene, DQ831586; bacterial 16S rRNA gene, FJ656473, respectively) at abundances ranging from 10^3^ to 10^8^ gene copies per reaction. These curves were used to estimate gene abundance in the seawater samples. The r^2^ value for the standard curve was 0.99, and the slope value was −3.14, giving an estimated amplification efficiency of 93%. The specificity of real-time PCRs was tested by analyzing melting curves, checking the sizes of reaction products using gel electrophoresis, and sequencing of the reaction products [25].

### PCR amplification and pyrosequencing of the prokaryotic 16S rRNA genes

PCR amplifications of prokaryotic (bacterial and archaeal) 16S rRNA genes including the V4 and V8 hypervariable regions were done with the prokaryotic universal primers 787F (5′-ATTAGATACCCNGGTAG-3′) [26] and 1391R (5′-ACGGGCGGTGWGTRC-3′) primer set [27]. PCR was performed using 25 μl of 2× PCR Master Mix Solution (Intron, Seongnam, Korea), 1 μM of each primer (final concentration) and ca 10 ng of genomic DNA as the template, and water was added to a final volume of 50 μl. The following PCR cycles were used: initial denaturation at 94°C for 5 min. followed by 30 cycles of 94°C for 50 s, 55°C for 30 s and 72°C for 50 s and a final extension step at 72°C for 6 min. The amplification products from each sample were purified using a PCR purification Kit (Cosmo4, Seoul, Korea). The DNA was quantified using a spectrophotometer (Nanodrop Technologies, Rockland, DE, USA) and was then mixed in equivalent proportions. Sequencing was performed using an GS FLX Titanium Genome Sequencer (454 Life Sciences, Branford, CT, USA). Multiplex identifiers (Roche, Basel, Schweiz), an adaptor and a short four-nucleotide sequence (TCAG), which were recognized by the system software and the priming sequences, were used to label the end fragments of the amplification products obtained from the samples by a sequencing provider (Macrogen, Seoul, Korea) according to the manufacturer’s instructions.

### Sequence analysis

Additional processing of the 16S rRNA gene sequences was done with QIIME 1.9.1 [28]. Paired-end reads were aligned using the fastq-join algorithm from ea-utils [29, 30]. Non-paired reads were discarded. The resulting sequences were then filtered at a Phred score of 20. Chimeric sequences were identified and removed using the UCHIME algorithm in USEARCH [31]. Open-reference OTU picking was done with uclust against the Greengenes 13_8 database [32] with a 0.97 similarity cutoff. Singletons were removed as the part of the OTU picking process. Sequences identified as chloroplasts or mitochondria and OUT with <1% abundance were then removed from the resulting OTU table by filtering. OTU tables for taxonomic groups specific to *Archaea* including MGII were extracted from the resulting complete data. New alignments and tree files were generated through QIIME specific to the new OTU tables. Sequence data that support the findings of this study have been deposited in GenBank with the BioProject accession code PRJNA448352.

### Statistical analysis

All analyses were done in the R software package v. 3.3.1 [33] with appropriate packages (vegan, MASS, tree and ggplot2) and several custom scripts. Generalized linear modeling (GLM) was done for the 16S rRNA gene copy number and the Shannon diversity index (*H*’) of both the archaea and bacteria communities over a period of 1 year (February, April, July and October) from two different bays (GI and GR) with simultaneously measured environmental variables including physicochemical parameters (e.g., salinity, temperature, pH, etc.), environmental parameters (e.g., DOC, PON, NH_4_^+^, etc.) and biological parameters ([chl-*a*] and DO). The overall trends of the samples were assessed using non-metric multidimensional scaling (NMDS) ordination. Seasonal and spatial patterns were tested with permutational multivariate analysis of variance (PERMANOVA) and analysis of similarity (ANOSIM). Procrustes test with NMDS ordination configurations and the Mantel test were used to compare the archaeal and bacterial communities as well as the composite environmental variables. Redundancy analysis (RDA) models were constructed to describe the community structure ordination in linear environmental constraints following a similar procedure as that of the GLM for *H*’. Briefly, models were selected by a reduction approach from the full model based on multicollinearity (VIF) among the predictors (glm) and constraints (RDA) and by model comparison approaches based on information criterion (AIC and BIC). Environmental gradients were built on NMDS ordination using the vegan::ordisurf function which used generalized addictive modeling (GAM) to overlay environmental variables in the ordination space [34]. The temperature and nitrite concentration gradient were selected by vector fitting to the NMDS ordinations.

## Results

### Environmental parameters

**Fig 2:**
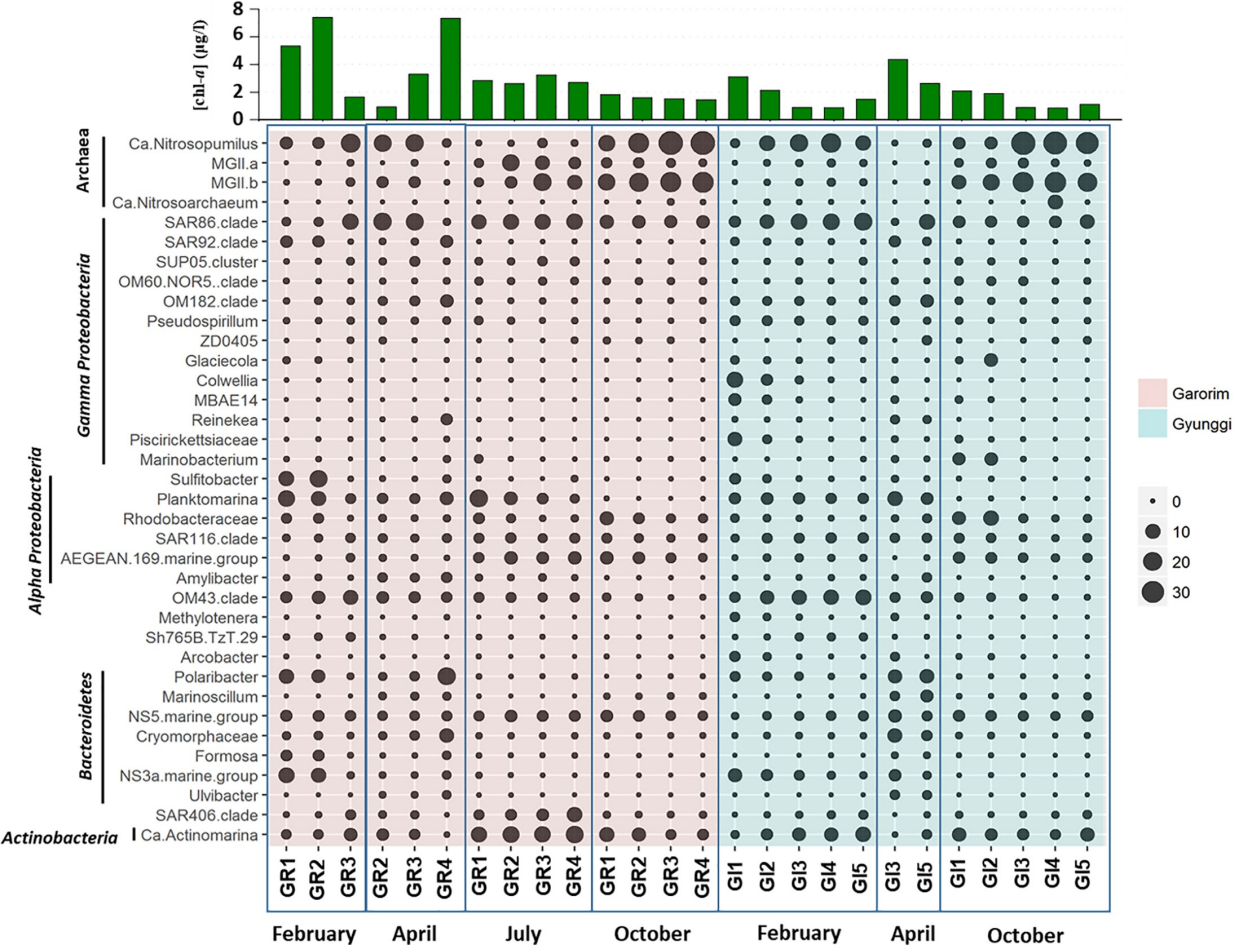
Taxonomic distribution of selected genera of dominant phyla of surface water microbiota in the two bays the Yellow Sea. [Chl-*a*] at each sample was shown at upper panel.

Salinity and other parameters at the GR stations (Table B in S1 Appendix) were more similar to each other due to a lack of freshwater inflow from land and the active tidal exchange of water with the open sea compared to those of GI stations (Fig 1). At the GI station, the salinity was lower at GI1 and GI2 where freshwater inflows from the Han River. The high SPM concentration was related to the tidal activity at both bays close to land and the inflow of the river at the GI stations. In addition, differences in other environmental factors such as pH, ammonia, and other nutrient concentrations support the influence of the freshwater inflows at GI1 and GI2. As appeared in Table B in S1 Appendix and Fig 2, the concentration of [Chl-*a*] shows strong spatial and seasonal variations. The winter (February) algal bloom was evident at the GR (GR1 and GR2) and GI (GI1 and GI2) stations close to land while the spring (April) algal bloom was observed at the GR (GR 3 and GR4) and GI (GI3 and GI5) stations close to ocean. It is interesting that during autumn (October), the nitrite concentration was especially high (1.5–4.2 μM) at the stations close to the open ocean such as GR3, GR4, GI3, GI4, and GI5.

### Archaeal and bacterial abundance and diversity

**Fig 3:**
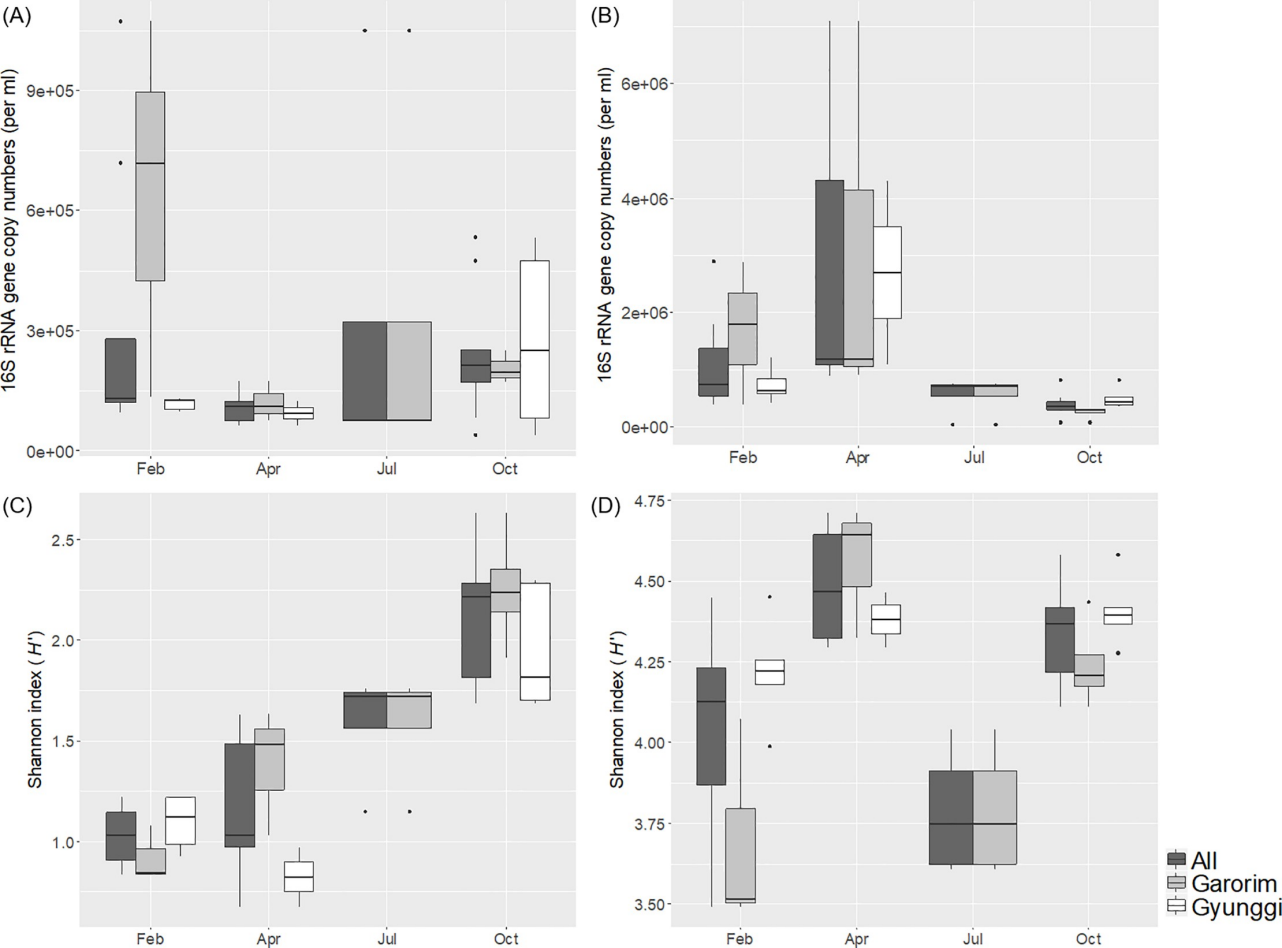
Box-whisker plots of abundance and diversity index per season for archaeal and bacterial assemblages measured by 16S rRNA gene qPCR and Shannon index (H’) of OTU counts. (A) Archaeal abundance, (B) Bacterial abundance, (C) Archaeal diversity and (D) Bacterial diversity.

The 16S rRNA gene copy number by qPCR and Shannon diversity index (*H*’) of the operational taxonomic unit (OTU) obtained from pyrosequencing were used to estimate the abundance and diversity of the archaeal and bacterial communities, respectively (Figure A in S1 Appendix, Fig 3C and 3D, and Table C in S1 Appendix). The trend for the relative abundance of bacteria and archaea estimated by qPCR was mostly similar with that by pyrosequencing except for GR1 and GR2 in February. Bacterial abundance was higher than archaeal abundance in all of the February, April, and July samples. Unexpectedly, the archaeal abundance was comparable with the bacterial abundance in most of the October samples at both sites except for at GI1 and GI2 where seasonal freshwater influx existed (Figure A in S1 Appendix). In February, both the archaeal and bacterial abundance were significantly higher inside Garorim Bay (GR1 and GR2) (Figure A in S1 Appendix). The site variation diminished in the July and October samples. Archaeal abundance was shown to be slightly increasing throughout the year except for the February archaeal bloom in GR whereas the bacterial abundance showed a decreasing trend from the April bloom to October (Fig 3 and Figure A in S1 Appendix). Archaeal diversity at both bay stations showed a near-linear increase from February to October, and the highest archaeal diversity was observed at the GR stations in October (Fig 2 and Figure B in S1 Appendix). Bacterial diversities at the GI stations were relatively constant throughout the year while the GR stations showed a significantly higher diversity in April and October than in February and July.

**Fig 4:**
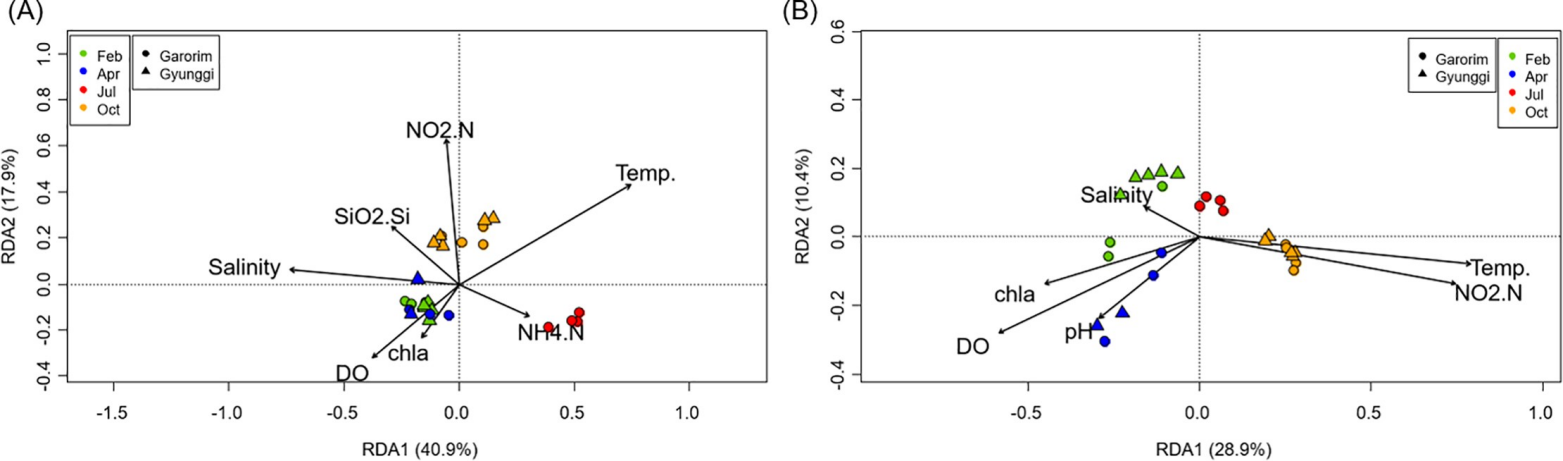
RDA models for archaeal (A. adj. R2 = 0.575, P = 0.001) and bacterial (B.adj.R2 = 0.410, P = 0.001) communities with selected significant environmental variables with minimum collinearity.

The trends for the abundance and diversity were fit by a generalized linear model (Figure C in S1 Appendix). As expected in the temporal trends shown in Fig 3 and Figure A in S1 Appendix, the archaeal diversity and bacterial abundance closely fitted a chronological seasonal pattern (February to October). The prime predictor of archaeal diversity was the temperature, which is a good proxy for the season. The best predictor for the bacterial abundance was [chl-*a*], as expected. The archaeal diversity and bacterial abundance were modeled very well with an adjusted coefficient of determination (*R*_adj_^2^) over 0.5 while the archaeal abundance and bacterial diversity regression models were fitted with a lower *R*_adj_^2^ (<0.2). Different parameters of the archaeal and bacterial communities were modeled with different environmental variables, and the temperature, NO_2_^-^, and [chl-*a*] were among the consistent predictors including community structure by a redundancy analysis (RDA) and by vector fitting (Fig 4 and Table D in S1 Appendix).

### Community composition and dynamics

The sequence reads were assigned to 50 described archaeal and bacterial phyla with five ubiquitous phyla making up an average of 94.9% of the reads: *Thaumarchaeota*, *Euryarchaeota*, *Proteobacteria*, *Bacteroidetes*, and *Actinobacteria* (Fig 2 and Figure D in S1 Appendix). The proportions of these five taxa in the surface seawater samples from the bay areas varied across the months; however, *Proteobacteria* were most abundant on average comprising 15%–77% of the sequence reads in all seawater samples. *Alpha-* and *Gamma-proteobacteria* were the most abundant subphyla of the *Proteobacteria*, and the relative abundance of these groups decreased in the October samples for all but the GI1 and GI2 stations. The second most abundant group on average was *Thaumarchaeota*, which showed the opposite temporal distributional pattern to *Proteobacteria* in that the relative abundance of *Thaumarchaeota* remained relatively low from February to July and became dominant in October. The relative abundance of *Euryarchaeota*, in particular MGIIb, increased during July and October. *Bacteroidetes* were abundant during February at the GR1 and GR2 stations and in April at the GR4, GI3, and GI5 stations. *Actinobacteria* and *Marinomicrobia* (SAR406 clade) were abundant in July at the GR stations.

Although strong temporal variation was the overall trend, several taxa were identified to have clear site variations (Fig 2). At the phyla level, *Alpha-proteobacteria*, *Gamma-proteobacteria*, and *Bacteriodetes* have somewhat different patterns of relative abundance between the two bay areas. Several *Gamma-proteobacteria* taxa such as *Glaciecola*, *Colwellia*, MBAE14, *Piscirickettsiaceae*, and *Marinobacterium* were more abundant at the GI stations, especially at GI1 and GI2, which have a large freshwater influx. On the other hand, some taxa belonging to *Alpha-proteobacteria* and *Bacteroidetes*, such as *Sulfitobacter*, *Planktomarina*, *Formosa*, and NS3a marine group, were more abundant at the GR stations. Some taxa at GI1 and GI2 appear to be linked to the geochemical dynamics of the freshwater influx compared with the other sampling stations of the GI area: 1) Several bacterial taxa such as *Colwellia*, MBAE14, *Piscirickettsiaceae*, *Sulfitobacter*, *Acrobacter*, *Polaribacter*, and NS3a marine group had a higher relative abundance in the February samples while the *Glaciecola* and *Marinobacterium* relative abundance was higher in the October samples; 2) Relative abundance of the other taxa including an archaeal taxon *Ca*. Nitrosopumilus was lower in both February and October; and 3) Bacterial taxa SAR86 clade, OM43 clade, NS5 marine group, and *Ca*. Actinomarina showed a lower relative abundance in February.

Because [chl-*a*] was the prime predictor of overall bacterial abundance (Figure C in S1 Appendix), some taxa showed a clear correlation with [chl-*a*]. In GR, significant positive correlations (α < 0.05) were found between [chl-*a*] and the relative abundances of taxa such as *Rhodobacteraceae*, *Sulfitobacter*, NS3a marine group, *Glaciecola*, NS5 marine group, and Formosa. The SUP05 cluster was negatively correlated with [chl-*a*]. In GI, significant positive correlations were found with [chl-*a*]: 1) *Alpha-proteobacteria* (*r* = 0.832, *P* < 0.001) and sub-taxa *Sulfitobacter* and SAR116 clade, 2) *Gamma-proteobacteria* (*r* = 0.760, *P* = 0.004) and sub-taxa *Pseudospirillum*, MBAE14, *Colwellia* and *Piscirickettsiaceae*, 3) *Beta-proteobacteria* (*r* = 0.647, *P* = 0.023) and sub-taxa *Methylotenera* and OM43 clade. Phylum *Chloroflexi* was found to be negatively correlated with [chl-*a*] (*r =* -0.700, *P* = 0.012).

### Community structure and environmental factors

**Fig 5:**
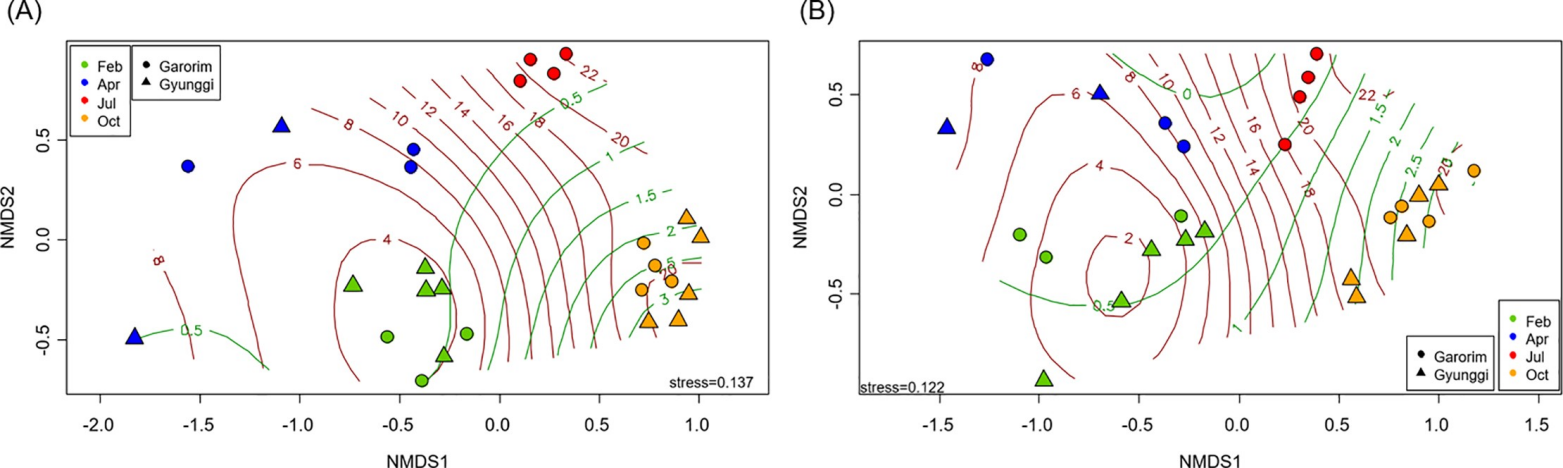
**NMDS ordination of archaeal (A) and bacterial (B) communities with Bray-Curtis distance.** Contour lines show a smooth generalized addictive model (GAM) surface reflecting best fitting environmental factors (Deviance explained: A. temperature 98.6% and NO_2_^-^ 88.4%, and B. temperature 96.8% and NO_2_^-^ 89.4% with P < 0.001 for all parameters).

Both archaeal and bacterial communities were distinctively clustered by season on the NMDS ordination (Fig 5) and tested by both ANOSIM and PERMANOVA (*P* < 0.001) (Table 1). The site distinction was shown to be not sufficiently clear. The composite environmental factors showed similar patterns to the archaeal and bacterial communities with greater overlap among the February to July samples (Figure E in S1 Appendix and Table 1). The Procrustes test and Mantel test both showed a significant association between the microbial communities and the composite environmental factors (Table E in S1 Appendix). Environmental factors were superimposed on the ordination space using generalized addictive model (GAM) surface contour instead of linear vectors for better representation of the non-linear nature of the relationship. Being top two among all environmental factors measured, the temperature and NO_2_^-^ concentration gradients fitting the ordinations of both archaeal and bacterial communities (Fig 5) explained over 96.8% (temperature) and 88.4% (NO_2_^-^) of the deviance with a *P* < 0.001.

**Table 1:** Permutation-based MANOVA-like analyses.

		Environmental parameters		*Archaea*		*Bacteria*	
		Month	Site	Month	Site	Month	Site
**PERMANOVA**	*F*	3.078	1.332	7.007	1.074	7.189	1.051
	*P*	0.002	0.246	<0.001	0.367	<0.001	0.370
**ANOSIM**	*R*	0.213	0.018	0.653	0.010	0.798	-0.006
	*P*	0.007	0.287	<0.001	0.352	<0.001	0.426

The RDA ordinations of the archaeal and bacterial communities were quite similar (*t* = 0.641, *P* < 0.001), but the RDA models built with environmental factors were quite distinctive. Archaeal communities were well explained by salinity, temperature, DO, NH_4_^+^, NO_2_^-^, SiO_2_, and [chl-*a*] with 58.8% explainable variance by the first two RDA axes. Total explainable variance was 69.4%, and *R*_adj_^2^ is 0.575. Temperature, salinity, and NO_2_^-^ were the top three environmental variables for the explainable variances. The February and April communities were largely inseparable and mostly controlled by DO, [chl-*a*], and salinity. The archaeal communities in July were mostly associated with NH_4_^+^ while October communities were associated with NO_2_^-^ and SiO_2_. The bacterial community RDA model was built with salinity, temperature, pH, DO, NO_2_^-^, and [chl-*a*] with 39.7% explainable variance by the first two axes, which is less than the archaeal communities. Total explainable variance (60.4%) and *R*_adj_^2^ (0.449) were also lower than those of the archaeal communities. Temperature and NO_2_^-^ were the top two environmental variables for the explainable variances. Bacterial communities were well separated per season and had distinctive environmental factors associated with them: salinity for February, pH, DO, and [chl-*a*] for April, and NO_2_^-^ and temperature for the October samples. No environmental parameter was associated with the July bacterial communities.

## Discussion

### Strong seasonal variations

We investigated the temporal dynamics of archaeal and bacterial communities of two bay areas on the west coast of the Korean Peninsula. While the surface seawater archaeal and bacterial communities showed strong temporal dynamics, they were largely indistinguishable between the two bay areas. The greater temporal variation of the microbial community was supported by the strong association with environmental factors, which showed greater temporal variations than the spatial variation as well as distinctive environmental factors associated with microbial communities for different seasons (Table 1 and Figure C in S1 Appendix). Stronger temporal patterns among microbial communities rather than locations were previously observed in the New Jersey coast and the Pearl River Estuary area [35, 36].

The temporal trends for the abundance and diversity of the archaeal and bacterial communities in these two bay areas were distinct. The abundance measured by the 16S rRNA gene copy number indicated a higher average bacterial abundance than archaeal abundance, consistent with other observations [37, 38]. The diversity was estimated by calculating the Shannon index (*H*’) from the OTU counts, which overall showed quite distinctive patterns compared with the 16S rRNA gene copy numbers for the abundance. Here, the comparisons we attempted to make were within each domain of the measurements and thus the difference in the abundance and diversity measures should not cause any notable problems.

### Bacterial community variation

We identified the spring bloom of bacterial communities at most sampling stations, which were observed in many other previous studies as well [39–42]. Bacterial abundance was best modeled with temperature, pH, and [chl-*a*], among which pH and [chl-*a*] were more specifically associated with the bacterial communities. Bacterial abundance was higher in the April samples from both bay areas as well as the diversity (Fig 3D and Figure A in S1 Appendix). The correlated trends between abundance and diversity indicated that the spring bacterial bloom was a more community-wide phenomenon because the taxa-specific results indicate most bacterial taxa; however, most prominently, *Bacteriodetes* had an increased abundance in both bay areas. An algal bloom often drastically reduce the diversity because only a few taxa of cyanobacteria and eukaryotic algae dominate the ecosystem with the opportunity given by the input of a large quantity of nutrients and warm temperature [43, 44]. Further, in this study cyanobacteria might be underestimated because of the low binding efficiency of PCR primers with the cyanobacterial 16S rRNA gene, as suggested by Bowman and colleagues (2012).[45] Although the algal bloom was observed in April as indicated by a higher [chl-*a*], the abundant *Proteobacteria* and several newly abundant *Bacteriodetes* taxa (e.g., *Polaribacter*, *Marinoscillum*, NS5 marine group, and *Cryomorphaceae*) appeared to consistently diminish the dominating effect by algae, and thus produced the opposite trend of a higher diversity by a higher evenness (*J*’_Apr._ = 0.827 and *J*’_Feb._ = 0.754) compared to the February samples with a comparable abundance. In the October samples, however, bacterial diversity was high while the abundance was low, potentially due to these samples having the second highest evenness (*J*’_Oct._ = 0.802) and a comparable richness (*S*_Oct._ = 1,316) to the other seasons (*S*_All_ = 1,445).

The temporal variation of the bacterial community composition is often driven by a few major phyla. From the current study, *Bacteroidetes* and *Alpha-proteobacteria* lead the high bacterial abundance during high [chl-*a*] seasons. *Bacteriodetes* (mainly *Polaribacter*) were dominant in February and April in both bay samples, especially when [chl-*a*] was high. Two dominant subphyla, *Alpha-proteobacteria* and *Gamma-proteobacteria*, had almost complementary relative abundance in different seasons, resulting in the consistently high abundance of *Proteobacteria* from February to July. *Alpha-proteobacteria* were more dominant at the GR stations mostly by two genera: *Sulfitobacter* and *Planktomarina*. *Sulfitobacter* and *Planktomarina* of the *Roseobacter* group have been shown to have close associations with a high [chl-*a*] [46–48]. Thus, it has been shown as a predominant community member after the peak of a bloom along with other members of the *Rhodobacteriaceae* family, as observed in the succession in nutrient-enriched marine mesocosms [49]. On the other hand, *Gamma-proteobacteria* had a higher relative abundance at the GI sites, mostly due to the SAR86 clade. Dominance of SAR86 in *Gamma-proteobacteria* year-round is unique in this area [50, 51].

### Archaeal bloom

The archaeal assemblages showed a particularly strong temporal trend in these areas. While the archaeal bloom in the February samples from the GR stations had the lowest diversity, the archaeal bloom in the October samples had a higher diversity (Fig 3C and Figure B in S1 Appendix). A sudden increase in the abundance may result in either increased or decreased diversity dependent on the community composition and dynamic interactions in association with niche availability [52, 53]. Because the diversity index calculation incorporates both richness and evenness of a community, the relative importance of the diversity components can determine the overall diversity index. The October samples had the highest richness with comparable evenness among the four sampling seasons. In other words, both the *Ca*. Nitrosopumilus and MGIIb group became overly dominant (Fig 2), and the MGIIa group and *Ca*. Nitrosoarchaeum became more abundant in October than in February and April. In contrast, when a community is dominated by a few opportunistic taxa (i.e., *r* strategists), the overall diversity index may decrease because the evenness would drastically decrease, as was the case in the current study (Shannon evenness *J*’_Feb.GI_ = 0.408). Both the October and February samples were overwhelmed by the *Ca*. Nitrosopumilus genus alone. While most inorganic nutrients were high in both the October and February samples, the temperature was much higher in October (20°C vs. 3.6°C), which has a general positive effect on metabolic activities, thus potentially encouraging more community-wide growth. The high input of inorganic nutrients in the GI samples in February may have promoted those fast-growing populations and thus, overall, reduced the diversity in both the archaeal and bacterial communities. Liu et al. (2018) suggested that SPM has a significant effect on the spatial distribution of *Ca*. Nitrosopumilus because of its relatedness to light penetration [54, 55]. In this study, however, the correlation between the composition of *Thaumarchaeota* and the SPM was weak, which suggests that other environmental factors may be controlling their distributions in our samples.

The relative abundance of both the *Euryarchaeota* (MGIIb group) and *Thaumarchaeota* (*Ca*. Nitrosopumilus genus) phyla overwhelmed all the other bacterial phyla in October (Fig 2), which was not common in other oceans. Furthermore, due to the single copy numbers of the 16S rRNA gene in the genomes of both the MGIIb group (*Euryarchaeota*) and *Ca*. Nitrosopumilus (*Thaumarchaeota*) [56, 57], the relative abundance by the 16S rRNA gene copy number of archaea was likely underestimated [58]. Overall, temperature and salinity were the most important predictors for the archaeal diversity and abundance, respectively. However, the pairwise correlation analysis did not reveal any significant correlations between the relative abundance of the four most abundant archaeal taxa and environmental factors except for a weak positive correlation between *Ca*. Nitrosoarchaeum and NO_2_^-^ (*r* = 0.401, *P* = 0.042). Note that a distinctive increase in October was observed in oxidized N species and PO_4_^3-^ concentrations (Table B in S1 Appendix). The environmental factors associated with the archaeal and bacterial communities per season were somewhat similar in that temperature, salinity, and oxidized N species were common for most measured aspects between them (Fig 5 and Table B in S1 Appendix). Of course, additional environmental factors were identified to be more specific to the archaeal community such as SiO_2_, SPM, DOC, and POC.

*Thaumarchaeota* is rarely found abundantly in the surface water of oceans and coastal seas [4, 39]. Nevertheless, we found that one of the *Ca*. Nitrosopumilus OTUs was dominant in the surface water of both bay areas. The high nitrite accumulation in the October samples may be associated with the high *Ca*. Nitrosopumilus abundance (*r* = 0.769, *P* = 0.015). One of the *Ca*. Nitrosopumilus OTUs was dominant at both bay areas. Furthermore, sequences for ammonia-oxidizing bacteria and nitrite-oxidizing bacteria were not detected at the study sites. Although the primary nitrite maximum has been suggested to be caused by algal nitrite release [59], in this study, a low [chl-*a*] and high *Ca*. Nitrosopumilus abundance (*r =* -0.880, *P* = 0.002) suggest archaeal ammonia oxidation to be a major cause of nitrite accumulation. High nitrite accumulation as observed in this study is unusual [60, 61], and may be caused by an imbalance of rates between ammonia production and nitrite oxidation.

It is interesting that MGIIb bloom is co-occurring with *Ca*. Nitrosopumilus bloom in the October samples from both bay areas (Fig 2). There were several reports of MGII bloom in oceans, and most were coastal areas. A time-series assessment of planktonic archaea in the Santa Barbara Channel revealed blooms of MGII coinciding with decreases in [chl-*a*] [62]. Another seasonal study of the surface water at the German Bight in the North Sea showed a spring bloom of MGII having >30% of the total cell counts and > 90% of all archaeal cells [63]. Galand et al. [64] speculated that the greater abundance of MGII in the Arctic Ocean may be related to the higher availability of labile organic matter from land surrounding the Arctic. In another study on the surface water of the Western Arctic, *Euryarchaeotal* abundance was very low throughout the year [65, 66] which lacked direct river inputs [67]. In contrast, in the Gyeonggi Bay area, the GI1 and GI2 stations, which were the most highly influenced by river inputs, showed a lesser archaeal abundance in both February and October. Our results coincide with the observation that brackish waters contain less archaea than seawaters [36], indicating that river inputs may not be a direct cause of archaeal blooms at these bays. The MGII group might be involved in the degradation of POC including proteins [57, 68, 69]; thus, ammonification by MGII might provide ammonia for nitrification by ammonia-oxidizing archaea, such as *Ca*. Nitrosopumilus, suggesting a co-dominance and functional coupling between the two archaeal clades.

### Site-specific variations

Overall, the archaeal and bacterial community composition showed very clear temporal variations, but several taxa showed a certain degree of site variations between the two bay areas as well (Fig 2 and S3 Figure D in S1 Appendix). The changes in relative abundances of several site-specific taxa in the GI1 and GI2 station samples were significant enough to cause substantial compositional changes at the phyla (Figure D in S1 Appendix) and OTU (Figure E in S1 Appendix) level. There were several environmental factors clearly associated with the GI1 and GI2 samples that might be direct results of a large quantity of freshwater influx: a low salinity and DO and high DIN, SPM, and POC in both the February and October samples (Table B in S1 Appendix). The site-specific taxa were not phylogenetically clustered because they spread across all phyla. With a limited number of samples, qualitative observation for the composition and relative relationship on the ordination space was attempted. NMDS achieves its configuration by positioning observations and variables into an *a priori* determined number of dimensions and preserving their relative distances among each other. The nice clustering between GI1 and GI2 and the separation from the other station samples thus ensured community distinction. Many of those taxa such as *Gamma-proteobacteria* with an altered relative abundance at the GI1 and GI2 sites might be attributable to the high freshwater influx.

In conclusion, we investigated the spatio-temporal dynamics of archaeal and bacterial communities at two geographically close bay areas on the west coast of the Korean Peninsula. Dynamic temporal changes in environmental factors observed at each station were associated with the variations in the archaeal and bacterial assemblages. The prime predictors of archaeal diversity and bacterial abundance were temperature and [chl-*a*], respectively. Temperature, NO_2_^-^, and [chl-*a*] were important environmental variables for both archaea and bacteria communities. Active community dynamics and population interactions resulted in complementary dominance and interesting trends in the abundance and diversity between the archaeal and bacterial populations. The archaea of the MGIIb group and *Ca*. Nitrosopumilus were co-dominant in October. There were also clear site-specific variations, especially at the stations under a freshwater influx. The distinct dynamics of the archaeal and bacterial assemblages observed in this study localized to coastal oceans were greatly affected by tides and nutrient-rich water inputs. Taken together, the results of this study provide insight into the dynamics and potential role of biogeochemistry in archaeal and bacterial communities in coastal surface seawater.

## Supporting information

S1 Appendix Supplementary results. **Figure A. Abundance of archaea and bacteria in Garolim and Gyeonggi Bays measured by 16S rRNA gene qPCR. Figure B. Box-whisker plots of abundance and diversity index per bay for archaeal and bacterial assemblages measured by 16S rRNA gene qPCR and Shannon index (H’) of OTU counts.** (A) Archaeal abundance, (B) Bacterial abundance, (C) Archaeal diversity and (D) Bacterial diversity. **Figure C. Archaeal and bacterial abundance and diversity index along the best predictor environmental parameter fitted by generalized linear modeling (GLM).** (A) Archaeal abundance with salinity, *R*_adj_^2^ = 0.190, (B) Bacterial abundance with [chl-*a*], *R*_adj_^2^ = 0.743, (C) Archaeal diversity with temperature, *R*_adj_^2^ = 0.572 and (D) Bacterial diversity with NO_2_^-^-NO_3_^-^, *R*_adj_^2^ = 0.160. **Figure D. Taxonomic distribution of the dominant phyla of surface water microbiota in Garolim and Gyeonggi Bays. Figure E**. **NMDS ordination of composited environmental parameters with Bray-Curtis distance. Table A. Sampling site. Table B. Environmental parameters in the sampling site. Table C. Sequence reads, richness and diversity index.** *S*: observed richness, *H*’: Shannon index. **Table D. Multiple regression, vector fitting and RDA results.** Bold in multiple regression predictors indicates the most important one determined by approaches in realimpo package in R. Vector fitting was done against NMDS ordination configuration using vegan::envfit and selected with P < 0.05. Bold font indicates the prime predictor in GLM models (Figure C S1 Appendix). **Table E. Procrustes and Mantel test results.**
(DOCX)
