# Supplementary material for: Distinct temporal dynamics of planktonic archaeal and bacterial assemblages in the bays of the Yellow Sea
Source: PLoS One. 2019 Aug 26;14(8):e0221408. doi: 10.1371/journal.pone.0221408 (PMC6709916; doi:10.1371/journal.pone.0221408)
Supplement: S1 Appendix — Figure A. Abundance of archaea and bacteria in Garolim and Gyeonggi Bays measured by 16S rRNA gene qPCR. Figure B. Box-whisker plots of abundance and diversity index per bay for archaeal and bacterial assemblages measured by 16S rRNA gene qPCR and Shannon index (H’) of OTU counts. (A) Archaeal abundance, (B) Bacterial abundance, (C) Archaeal diversity and (D) Bacterial diversity. Figure C. Archaeal and bacterial abundance and diversity index along the best predictor environmental parameter fitted by generalized linear modeling (GLM). (A) Archaeal abundance with salinity, Radj2 = 0.190, (B) Bacterial abundance with [chl-a], Radj2 = 0.743, (C) Archaeal diversity with temperature, Radj2 = 0.572 and (D) Bacterial diversity with NO2--NO3-, Radj2 = 0.160. Figure D. Taxonomic distribution of the dominant phyla of surface water microbiota in Garolim and Gyeonggi Bays. Figure E. NMDS ordination of composited environmental parameters with Bray-Curtis distance. Table A. Sampling site. Table B. Environmental parameters in the sampling site. Table C. Sequence reads, richness and diversity index. S: observed richness, H’: Shannon index. Table D. Multiple regression, vector fitting and RDA results. Bold in multiple regression predictors indicates the most important one determined by approaches in realimpo package in R. Vector fitting was done against NMDS ordination configuration using vegan::envfit and selected with P < 0.05. Bold font indicates the prime predictor in GLM models (Figure C S1 Appendix). Table E. Procrustes and Mantel test results. (DOCX) [file pone.0221408.s001.DOCX]

**S1 Appendix: Figures and Tables**

**Supplementary Figures**

**Figure A**. Abundance of archaea and bacteria in Garolim and Gyeonggi Bays measured by 16S rRNA gene qPCR.


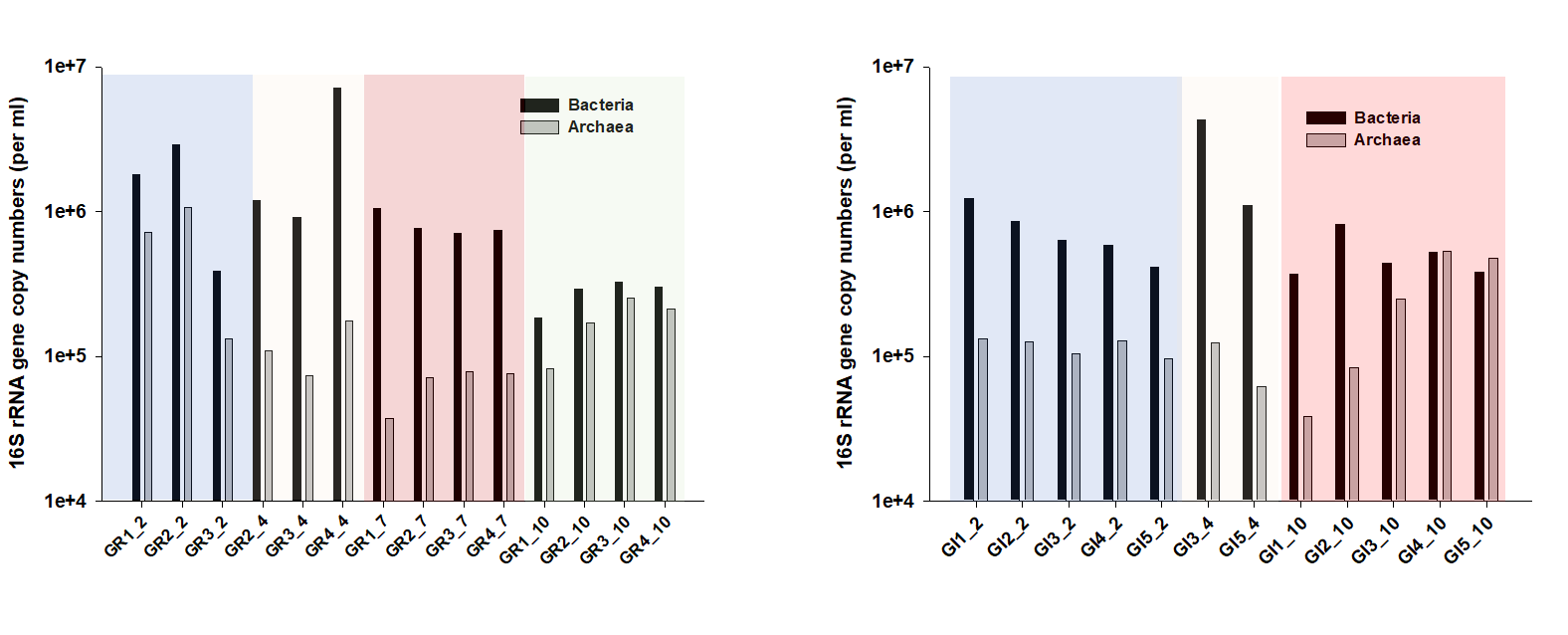


**Figure B**. Box-whisker plots of abundance and diversity index per bay for archaeal and bacterial assemblages measured by 16S rRNA gene qPCR and Shannon index (*H*’) of OTU counts. (A) Archaeal abundance, (B) Bacterial abundance, (C) Archaeal diversity and (D) Bacterial diversity.


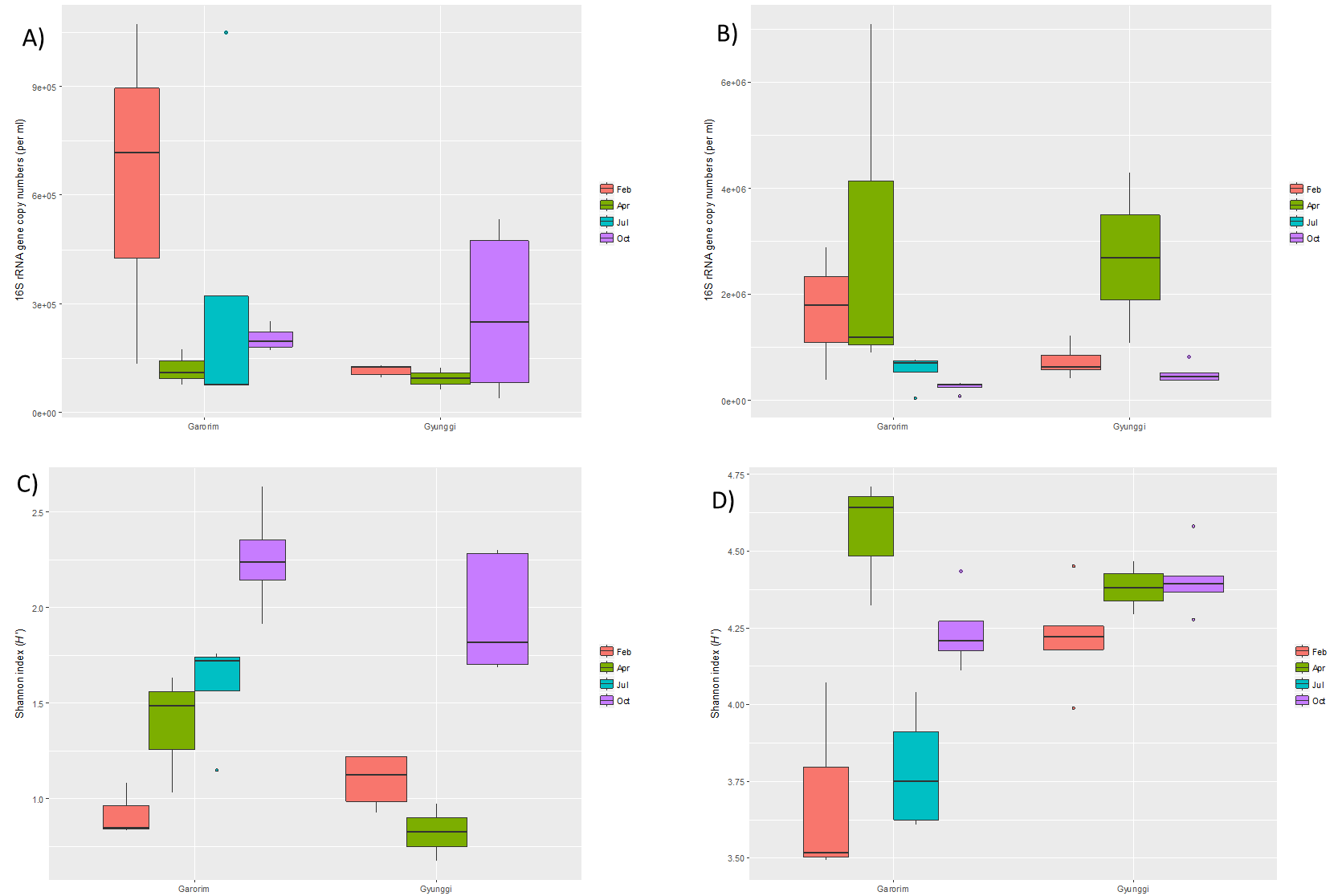


**Figure C**. Archaeal and bacterial abundance and diversity index along the best predictor environmental parameter fitted by generalized linear modeling (GLM). (A) Archaeal abundance with salinity, *R*_adj_^2^ = 0.190, (B) Bacterial abundance with [chl-*a*], *R*_adj_^2^ = 0.743, (C) Archaeal diversity with temperature, *R*_adj_^2^ = 0.572 and (D) Bacterial diversity with NO_2_^-^-NO_3_^-^, *R*_adj_^2^ = 0.160.


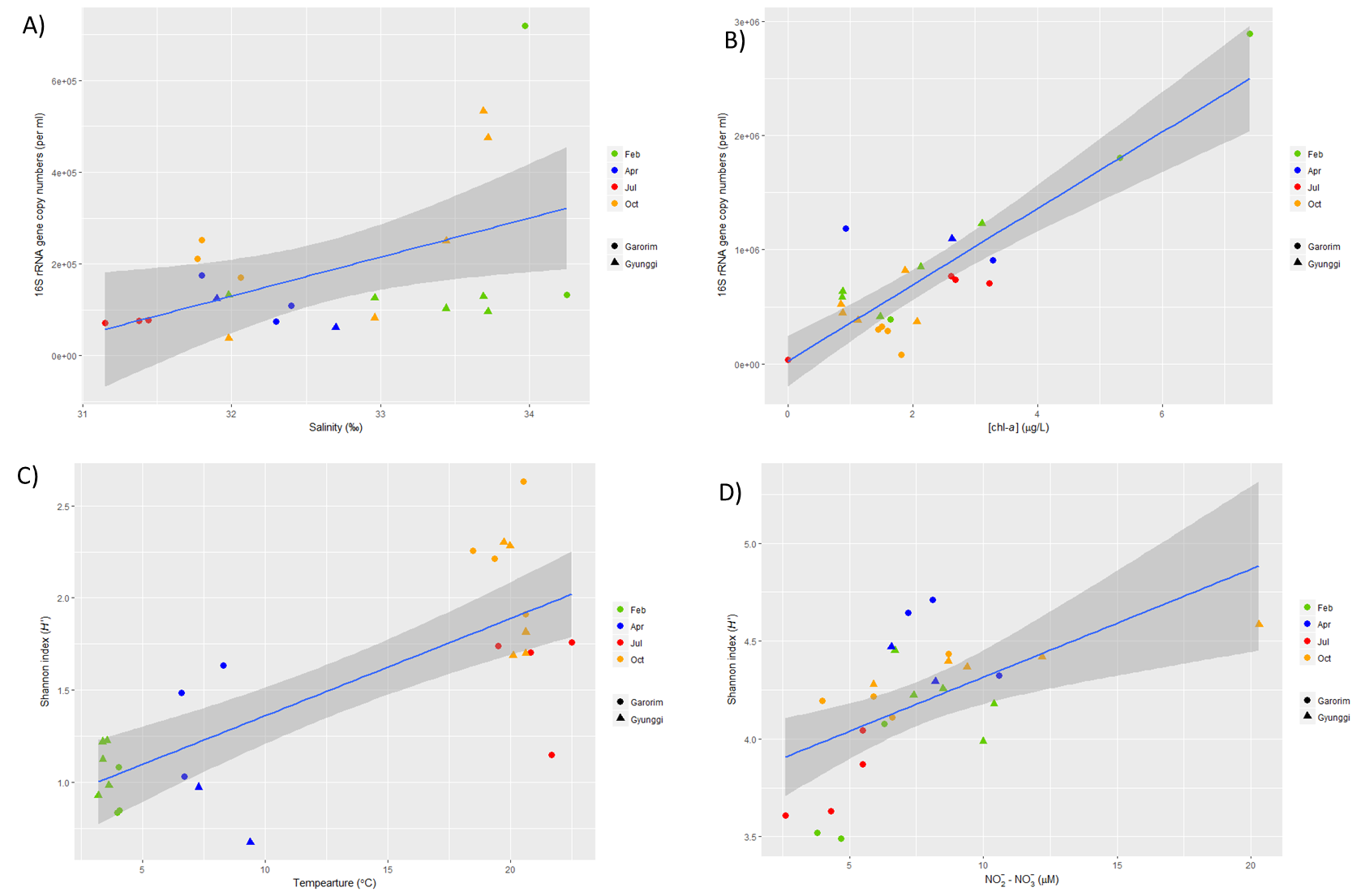


**Figure D**. Taxonomic distribution of the dominant phyla of surface water microbiota in Garolim and Gyeonggi Bays.


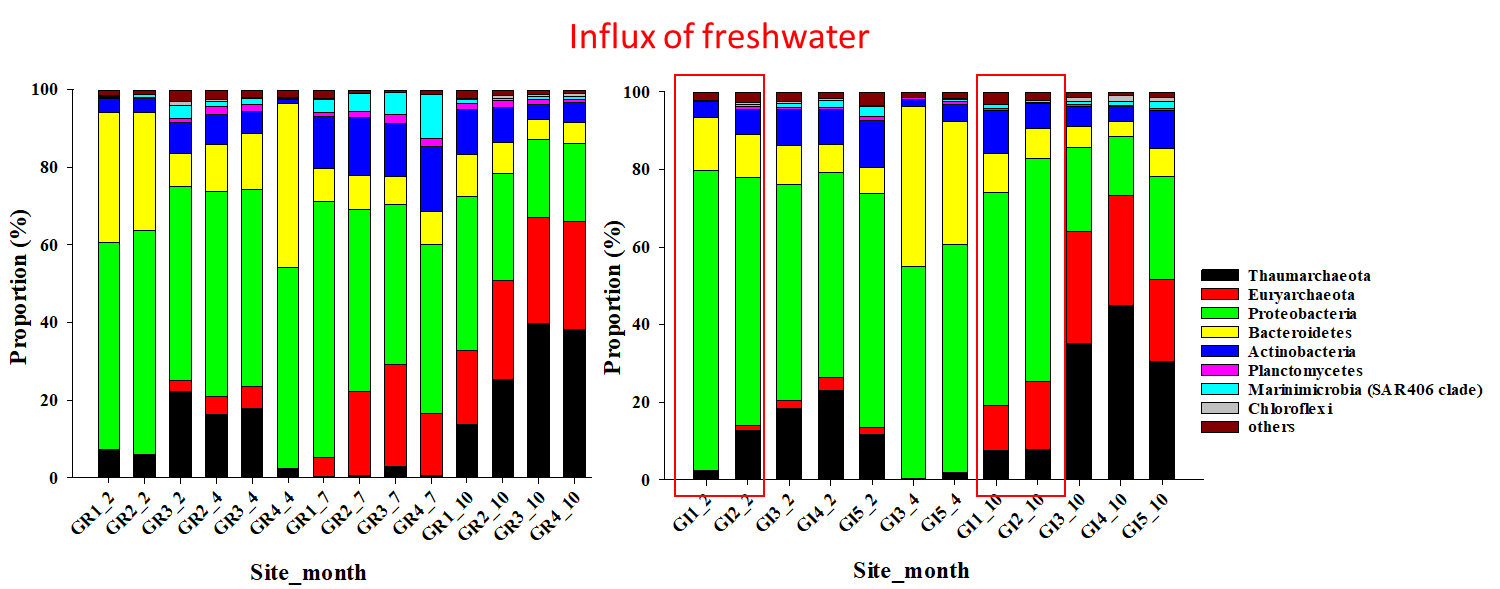


**Figure E**. NMDS ordination of composited environmental parameters with Bray-Curtis distance.

**Table A**. Sampling site

| **Sampling site** | **Latitude**  **(°N)** | **Longitude**  **(°E)** |  | **Sampling site** | **Latitude**  **(°N)** | **Longitude**  **(°E)** |
| --- | --- | --- | --- | --- | --- | --- |
| **GR1** | 36°53'14.52" | 126°21'13.48" |  | **GI1** | 37°34'59.95" | 126°34'11.39" |
| **GR2** | 36°55'32.00" | 126°20'21.84" |  | **GI2** | 37°25'52.63" | 126°33.53.90" |
| **GR3** | 37°1'51.02" | 126°17'41.91" |  | **GI3** | 37°21'31.43" | 126°30'55.00" |
| **GR4** | 37° 7'40.55" | 126°32'20.49" |  | **GI4** | 37°19'08.76" | 126°20'06.31" |
|  |  |  |  | **GI5** | 37°15'49.89" | 126°13'43.39" |

**Table B**. Environmental parameters in the sampling site.

|  | **Station** | **Salinity** (PSU) | **Temp.** (°C) | **pH** | **DO** (mg/l) | **SPM** (mg/l) | **DOC** (µM) | **POC** (µM) | **PON** (µM) | **NH_4_-N** (µM) | **NO_2_-N** (µM) | **NO_2_+NO_3_-N** (µM) | **NO_3_-N** (µM) | **DIN** (µM) | **PO_4_-P** (µM) | **SiO_2_-Si** (µM) | **Chla** (µg/l) |
| --- | --- | --- | --- | --- | --- | --- | --- | --- | --- | --- | --- | --- | --- | --- | --- | --- | --- |
| **Feb** | **GR1** | 33.97 | 3.97 | 8.5 | 11.88 | 7.0 | 118 | 24 | 1.7 | 2.6 | 0.29 | 4.7 | 4.4 | 7.3 | 0.10 | 9.3 | 5.32 |
|  | **GR2** | 34.06 | 4.06 | 8.48 | 11.35 | 8.7 | 110 | 29 | 2.1 | 1.1 | 0.43 | 3.8 | 3.3 | 4.8 | 0.13 | 8.4 | 7.40 |
|  | **GR3** | 34.25 | 4.03 | 8.33 | 11.08 | 41.1 | 100 | 54 | 2.4 | 0.6 | 0.33 | 6.3 | 6.0 | 6.9 | 0.23 | 9.1 | 1.64 |
|  | **GI1** | 31.98 | 3.38 | 8.25 | 6.57 | 25.8 | 129 | 48 | 2.3 | 7.4 | 0.80 | 10.4 | 9.6 | 17.75 | 0.22 | 7.9 | 3.11 |
|  | **GI2** | 32.96 | 3.2 | 8.3 | 6.45 | 41.9 | 135 | 79 | 2.0 | 2.9 | 0.44 | 6.7 | 6.3 | 9.67 | 0.26 | 8.0 | 2.13 |
|  | **GI3** | 33.44 | 3.39 | 8.31 | 7.18 | 6.4 | 121 | 28 | 1.5 | 2.9 | 0.42 | 8.5 | 8.1 | 11.43 | 0.38 | 9.7 | 0.88 |
|  | **GI4** | 33.69 | 3.56 | 8.31 | 6.86 | 2.6 | 110 | 19 | 2.6 | 2.2 | 0.41 | 10.0 | 9.6 | 12.17 | 0.46 | 13.0 | 0.87 |
|  | **GI5** | 33.72 | 3.63 | 8.31 | 6.77 | 13.1 | 107 | 23 | 2.3 | 0.9 | 0.35 | 7.4 | 7.0 | 8.30 | 0.35 | 11.8 | 1.48 |
| **April** | **GR2** | 32.4 | 6.7 | 8.4 | 10.2 | 13.5 | 136 | 34 | 0.4 | 4.1 | 0.19 | 10.6 | 10.4 | 14.7 | 0.4 | 8.90 | 0.93 |
|  | **GR3** | 32.3 | 6.6 | 8.2 | 10.6 | 19.9 | 111 | 37 | 1.4 | 4.5 | 0.15 | 7.2 | 7.1 | 11.7 | 0.3 | 5.05 | 3.29 |
|  | **GR4** | 31.8 | 8.3 | 8.2 | 11.6 | 5.0 | 137 | 23 | 2.0 | 5.3 | 0.29 | 8.1 | 7.8 | 13.4 | 0.0 | 0.95 | 7.32 |
|  | **GI3** | 31.9 | 9.4 | 8.3 | 10.5 | 28.4 | 138 | 137 | 2.1 | 5.0 | 0.48 | 8.21 | 7.7 | 13.2 | 0.0 | 20.9 | 4.36 |
|  | **GI5** | 32.7 | 7.3 | 8.5 | 10.5 | 17.9 | 116 | 73 | 9.0 | 3.6 | 0.15 | 6.57 | 6.4 | 10.1 | 0.1 | 5.2 | 2.63 |
| **July** | **GR1** | 31.04 | 22.5 | 8.03 | 7.09 | 34.8 | 135 | 74 | 2.2 | 8.0 | 0.33 | 5.5 | 5.2 | 13.5 | 0.16 | 11.3 | 2.83 |
|  | **GR2** | 31.15 | 21.67 | 8.06 | 7.54 | 10.9 | 106 | 53 | 1.7 | 3.9 | 0.22 | 4.3 | 4.1 | 8.2 | 0.12 | 5.6 | 2.61 |
|  | **GR3** | 31.44 | 19.5 | 8.11 | 8.19 | 6.5 | 127 | 28 | 0.9 | 4.6 | 0.35 | 5.5 | 5.1 | 10.1 | 0.11 | 4.5 | 3.23 |
|  | **GR4** | 31.38 | 20.81 | 8.09 | 7.75 | 8.8 | 185 | 74 | 3.1 | 2.5 | 0.15 | 2.6 | 2.5 | 5.2 | 0.04 | 4.2 | 2.69 |
| **Oct** | **GR1** | 32.1 | 18.47 | 8.27 | 7.55 | 23.6 | 110 | 27 | 3.9 | 0.8 | 1.51 | 4.0 | 2.5 | 4.8 | 0.58 | 10.4 | 1.82 |
|  | **GR2** | 32.06 | 19.34 | 8.28 | 6.6 | 12.4 | 127 | 28 | 2.4 | 1.6 | 2.36 | 5.9 | 3.5 | 7.5 | 0.70 | 12.3 | 1.60 |
|  | **GR3** | 31.8 | 20.53 | 8.28 | 7.28 | 9.8 | 229 | 31 | 2.0 | 1.1 | 3.20 | 6.6 | 3.4 | 7.8 | 0.71 | 7.9 | 1.51 |
|  | **GR4** | 31.77 | 20.62 | 8.29 | 7.18 | 6.1 | 120 | 14 | 7.2 | 1.2 | 4.24 | 8.7 | 4.5 | 10.0 | 0.87 | 10.4 | 1.45 |
|  | **GI1** | 31.98 | 19.72 | 8.25 | 6.57 | 25.8 | 129 | 48 | 2.3 | 7.6 | 1.49 | 20.3 | 18.8 | 27.9 | 1.21 | 11.3 | 2.07 |
|  | **GI2** | 32.96 | 19.98 | 8.3 | 6.45 | 41.9 | 135 | 79 | 2.0 | 6.6 | 1.91 | 12.2 | 10.2 | 18.7 | 1.53 | 10.8 | 1.88 |
|  | **GI3** | 33.44 | 20.11 | 8.31 | 7.18 | 6.4 | 121 | 28 | 1.5 | 3.1 | 3.50 | 9.4 | 5.9 | 12.5 | 1.07 | 9.4 | 0.88 |
|  | **GI4** | 33.69 | 20.62 | 8.31 | 6.86 | 2.6 | 110 | 19 | 2.6 | 1.6 | 3.80 | 8.7 | 4.9 | 10.3 | 0.88 | 9.6 | 0.85 |
|  | **GI5** | 33.72 | 20.61 | 8.31 | 6.77 | 13.1 | 107 | 23 | 2.3 | 1.1 | 2.08 | 5.9 | 3.8 | 7.0 | 0.60 | 7.1 | 1.12 |

**Table C**. Sequence reads, richness and diversity index. *S*: observed richness, *H*’: Shannon index

|  | **Station** | **Archaea** | | | **Bacteria** | | |
| --- | --- | --- | --- | --- | --- | --- | --- |
|  |  | **Reads** | ***S*** | ***H*’** | **Reads** | ***S*** | ***H*’** |
| **Feb** | **GR1** | 127 | 10 | 0.833 | 1843 | 177 | 3.491 |
|  | **GR2** | 85 | 11 | 0.846 | 1416 | 169 | 3.518 |
|  | **GR3** | 424 | 23 | 1.080 | 1274 | 192 | 4.076 |
|  | **GI1** | 21 | 7 | 1.219 | 1242 | 160 | 4.179 |
|  | **GI2** | 308 | 19 | 0.928 | 1743 | 243 | 4.452 |
|  | **GI3** | 424 | 20 | 1.122 | 1851 | 246 | 4.257 |
|  | **GI4** | 457 | 20 | 1.224 | 1491 | 196 | 3.988 |
|  | **GI5** | 508 | 22 | 0.983 | 2829 | 307 | 4.222 |
| **April** | **GR2** | 34 | 5 | 1.031 | 1425 | 221 | 4.324 |
|  | **GR3** | 636 | 18 | 1.485 | 1711 | 256 | 4.645 |
|  | **GR4** | 372 | 15 | 1.633 | 1345 | 239 | 4.711 |
|  | **GI3** | 5 | 2 | 0.673 | 910 | 191 | 4.294 |
|  | **GI5** | 38 | 6 | 0.972 | 1431 | 237 | 4.469 |
| **July** | **GR1** | 270 | 19 | 1.759 | 4003 | 354 | 4.044 |
|  | **GR2** | 507 | 13 | 1.147 | 1276 | 154 | 3.630 |
|  | **GR3** | 840 | 20 | 1.737 | 1183 | 155 | 3.869 |
|  | **GR4** | 283 | 14 | 1.702 | 1060 | 146 | 3.609 |
| **Oct** | **GR1** | 1031 | 42 | 2.258 | 1744 | 253 | 4.195 |
|  | **GR2** | 1739 | 63 | 2.214 | 1478 | 250 | 4.218 |
|  | **GR3** | 578 | 44 | 2.633 | 596 | 148 | 4.110 |
|  | **GR4** | 2476 | 53 | 1.912 | 937 | 200 | 4.436 |
|  | **GI1** | 441 | 31 | 2.301 | 1934 | 295 | 4.583 |
|  | **GI2** | 892 | 39 | 2.280 | 1959 | 295 | 4.420 |
|  | **GI3** | 2039 | 60 | 1.687 | 1064 | 220 | 4.368 |
|  | **GI4** | 2701 | 70 | 1.814 | 686 | 180 | 4.395 |
|  | **GI5** | 1760 | 53 | 1.700 | 1368 | 218 | 4.277 |

**Table D.** Multiple regression, vector fitting and RDA results. Bold in multiple regression predictors indicates the most important one determined by approaches in realimpo package in R. Vector fitting was done against NMDS ordination configuration using vegan::envfit and selected with *P* < 0.05. Bold font indicates the prime predictor in GLM models (Fig. S4).

|  | | **Selected environmental parameters** | ***R*^2^_adj_** |
| --- | --- | --- | --- |
| **Archaea** | **Abundance** | **Salinity**, POC, NH_4_^+^, NO_3_^-^, SiO_2_ | 0.545 |
|  | **Diversity** | **Temperature**, SPM, DOC, POC | 0.811 |
|  | **Structure (RDA)** | Salinity, temperature, DO, NH_4_^+^, NO_2_^-^, SiO_2_, [chl-*a*] | 0.575 |
|  | **Structure (envfit)** | Salinity, temperature, pH, DO, NO_2_^-^, PO_4_^3-^, SiO_2_ |  |
| **Bacteria** | **Abundance** | Temperature, pH, **[chl-*a*]** | 0.809 |
|  | **Diversity** | Temperature, **NO_2_^-^- NO_3_^-^**, NO_3_^-^, POC, SiO_2_ | 0.345 |
|  | **Structure (RDA)** | Salinity, temperature, pH, DO, NO_2_^-^, PO_4_^3-^, [chl-*a*] | 0.449 |
|  | **Structure (envfit)** | Salinity, temperature, pH, DO, NO_2_^-^, PO_4_^3-^, [chl-*a*] |  |

**Table E**. Procrustes and Mantel test results.

|  | **Procrustes test** | | **Mantel test** | |
| --- | --- | --- | --- | --- |
|  | ***t*** | ***P*** | ***r*_M_** | ***P*** |
| **Archaea-environment** | 0.547 | 0.001 | 0.249 | 0.008 |
| **Bacteria-environment** | 0.526 | 0.001 | 0.248 | 0.004 |
| **Archaea-Bacteria** | 0.887 | < 0.001 | 0.656 | < 0.001 |
